# Toxicity Studies on Graphene-Based Nanomaterials in Aquatic Organisms: Current Understanding

**DOI:** 10.3390/molecules25163618

**Published:** 2020-08-09

**Authors:** Nemi Malhotra, Oliver B. Villaflores, Gilbert Audira, Petrus Siregar, Jiann-Shing Lee, Tzong-Rong Ger, Chung-Der Hsiao

**Affiliations:** 1 Department of Biomedical Engineering, Chung Yuan Christian University, Chung-Li 320314, Taiwan; nemi.malhotra@gmail.com; 2 Department of Biochemistry, Faculty of Pharmacy and Research Center for Natural and Applied Sciences, University of Santo Tomas, Manila 1015, Philippines; obvillaflores@ust.edu.ph; 3 Department of Chemistry, Chung Yuan Christian University, Chung-Li 320314, Taiwan; gilbertaudira@yahoo.com; 4 Department of Bioscience Technology, Chung Yuan Christian University, Chung-Li 320314, Taiwan; siregar.petrus27@gmail.com; 5 Department of Applied Physics, National Pingtung University, Pingtung 900391, Taiwan; 6 Center for Nanotechnology, Chung Yuan Christian University, Chung-Li 320314, Taiwan

**Keywords:** graphene, graphene oxide, fish, invertebrates, toxicity

## Abstract

Graphene and its oxide are nanomaterials considered currently to be very promising because of their great potential applications in various industries. The exceptional physiochemical properties of graphene, particularly thermal conductivity, electron mobility, high surface area, and mechanical strength, promise development of novel or enhanced technologies in industries. The diverse applications of graphene and graphene oxide (GO) include energy storage, sensors, generators, light processing, electronics, and targeted drug delivery. However, the extensive use and exposure to graphene and GO might pose a great threat to living organisms and ultimately to human health. The toxicity data of graphene and GO is still insufficient to point out its side effects to different living organisms. Their accumulation in the aquatic environment might create complex problems in aquatic food chains and aquatic habitats leading to debilitating health effects in humans. The potential toxic effects of graphene and GO are not fully understood. However, they have been reported to cause agglomeration, long-term persistence, and toxic effects penetrating cell membrane and interacting with cellular components. In this review paper, we have primarily focused on the toxic effects of graphene and GO caused on aquatic invertebrates and fish (cell line and organisms). Here, we aim to point out the current understanding and knowledge gaps of graphene and GO toxicity.

## 1. Introduction

Graphene is an allotrope of carbon [1]. It is defined as single layer of carbon sheet with a hexagonal packed lattice structure with *sp^2^* hybridized carbon atoms tightly packed in a 2D honeycomb lattice, which provides large surface area on both sides of the planar axis [2]. The unmodified basal plane sites of graphene comprise free surface π electrons that are hydrophobic [3]. However, the hydrophobicity of the graphene is strongly thickness-dependent, with single-layer graphene being significantly more hydrophilic than its thicker counterparts [4]. The ideal graphene is a single-layer carbon atom held together by covalent bonds. It is difficult to isolate a single layer of graphene, hence graphene is also categorized as few-layer graphene (2–5 nm), multilayer graphene (2–10 nm), and graphite nanoplates (2D graphite materials, thickness > 100 nm) [5,6].

**Figure 1:**
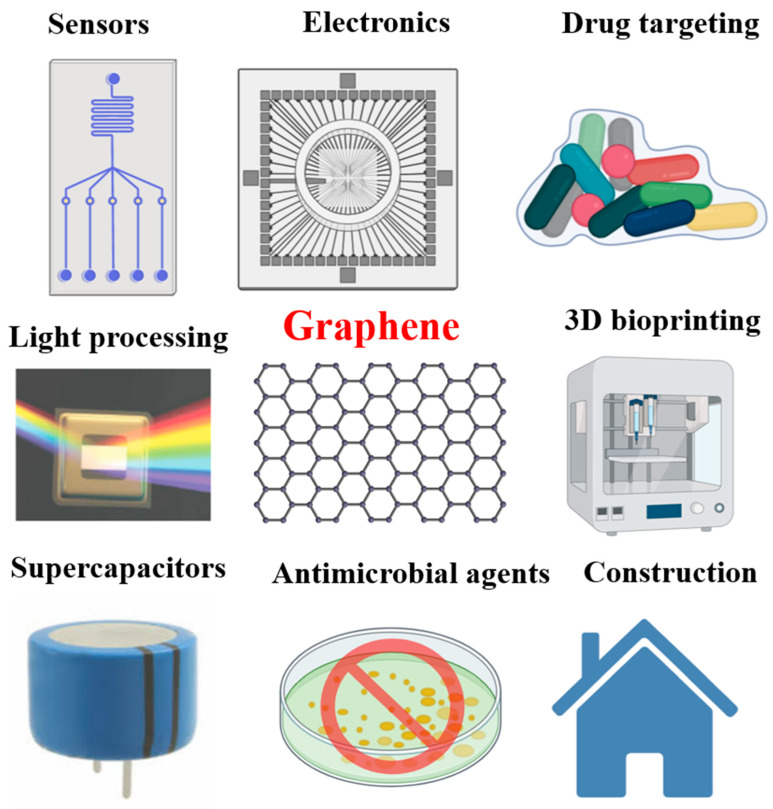
Potential application of graphene-based nanomaterials in industrial and biomedical fields. The graphene-based nanomaterials are widely applied for producing sensor, electronic, drug targeting, 3D bioprinting, construction, antimicrobial agents, supercapacitor, and light processing.

It is the strongest and thinnest known material known to man [7]. Graphene consists of unique properties such as thermal conductivity [8,9], impermeability to gases, stiffness, high Young’s modulus [9], good optical transparency [10], high carrier mobility at room temperature, excellent mechanical strength, large surface area, and outstanding electrocatalytic activity [1,2,7,11,12]. These unique and appealing properties of graphene make it potential candidate for biomedical application, including absorption of enzymes [13,14], electrochemical devices [15,16], energy storage [17,18], drug delivery [19], biosensors [20,21], photovoltaic devices [22,23], supercapacitors [24,25], photocatalysis [26,27], and fuel cells [1,2,19,28,29] (Figure 1).

These advantages of graphene make it desirable for the development of graphene composites through the incorporation of different functional materials. Graphene-based composites have been achieved successfully with organic crystals [30,31], inorganic nanostructure [32,33,34,35], biomaterials [21,36], polymers [37,38,39], and carbon nanotubes [40,41,42].

On the other hand, graphene oxide (GO) is an intermediate product obtained during the synthesis of reduced GO (rGO) [43,44,45]. It is also a two-dimensional (2D) carbon material and is viewed as the oxidized form of graphene, with oxygen functional groups decorating the basal plane of carbon layer [46]. It can be regarded as a result of chemical exfoliation and oxidizing of layered crystalline graphite [47]. Owing to the presence of the oxygen functional groups, GO nanosheets are hydrophilic in nature. They can be dispersed in aqueous solution [46] but are not dispersive in organic solvents. These attractive properties of GO make it suitable for biomedical applications such as gene delivery, drug delivery, scaffolds for mammalian cell culture, and substrates for antibacterial agents [48,49].

At this increasing phase of commercial production, the presence of graphene is evident in the environment at a significant level [50,51]. Irrespective of particular forms of graphene, a large number of studies have demonstrated that graphene affects a wide range of living organisms, including prokaryotes, bacteria, viruses, plants, micro and macroinvertebrates, mammals, human cells, and whole animals in vivo [52]. Further, graphene also interacts with physiological components of aquatic environment such as inorganic ions, colloidal particles, surface active molecules, and natural organic matter (NOM), which are known to modify graphene surface [6]. Thus, the interaction with the surrounding media and biota with graphene has demonstrated its transformation and degradation for possible bioremediation of graphene and its oxides [53]. In particular, Indranil Chowdhury et al. (2013) suggested that GO aggregation and stability in aqueous environment follows a colloidal theory [54]. GO contain many oxygen-containing groups, it is dispersible in water, and can be transported in water through physical process or food chain. As a result, it might accumulate in the ecosystem, posing threat for aquatic organisms and eventually to human health [55,56,57]. Recently, in vivo studies have revealed bio-distribution and persistence of GO in living organisms [58,59,60]. The hydrophobic lattice of graphene tends to undergo layer-by-layer aggregation in water due to hydrophobic forces, whereas GO with carboxyl, hydroxyl, and epoxy groups on the surface forms stable suspensions [54]. Many investigations have assessed the effects and mechanisms related to the transport and intake of nanoparticles in aquatic invertebrate and fish model at different growth stages to address the eco-toxicity [61,62,63]. Many biomarkers of environment toxicity notably the density of cellular and subcellular components of blood, lysosomal membrane stability, apoptosis, micronucleation, cellular damages, and cytotoxic responses have been established. Moreover, behavioral shifts and histopathological analyses are suggested as effective parameters of toxicity screening in model invertebrates [64]. On the other hand, fish is an important species in the aquatic food chain. Fish is potentially exposed to released nanoparticles to the environment via the food chain or by direct absorption/adsorption from aquatic medium [65]. The fish model includes sensitive early life-stage bioassays, sensitivity to dissolved chemicals and materials, low rearing cost, and homology with the human genome. The emphasis on fish model as an adjunct to conventional rodent models is gaining acceptability in toxicological research of environment contaminants, especially the ones having direct impact on aquatic systems [66]. Hence, results from thorough analyses of graphene-exposed fish might lead to some constructive outcome for future developments in research.

In this present review, we will analyze the biocompatibility and toxicology of graphene and GO in order to evaluate the safety limits needed for the implementation of upcoming researches. We will also try to draw attention towards knowledge gaps to attain valuable insights on the fate and risks presented by graphene and GO on fish in the aquatic environment. Furthermore, we will discuss the key physiological factors involved in influencing the toxicity of graphene and GO in fish in the surrounding water medium, degradation pathways, and the exposure and administration pathways. We believe that the critical study in this field will lead to collection of current information leading to the production of better modified materials emerging in healthcare, diagnostic, and therapeutics.

## 2. Graphene Chemistry

Graphene is inert in chemistry because of the immense delocalized π electron system [67]. The graphene in its pure form is hydrophobic and has very less solubility in most of the solvents. The chemistry of graphene is primarily focused on to analyze the solubilization of graphene, and various methods have been developed to attain its well-organized chemical modification. To increase the solubility of graphene, wet chemistry techniques have been devised that are also used for graphite and carbon nanotubes [45,68]. The graphene sheet can readily be functionalized via non-covalent p-p stacking or covalent C-C coupling reactions. In particular, covalent chemistry provides a strong pathway to tailor physical properties of pristine graphene. The carbon atoms present in graphene are chemically accessible. The derivation of graphene with different organic moieties makes graphene solubility flexible to be adjusted to different solvents required for the processing of composite films [69,70,71]. Furthermore, functional groups used for modification can broaden the properties of graphene through formation of donor-receptor complex with graphene. Based on the properties of graphene and its derivatives, significant movement in mechanical, thermal, electrical, and viscous properties of graphene-polymer composites can be achieved [72]. The aggregation and dispersion behavior of graphene thus can be altered by solution chemistry. GO demonstrates different dispersion performance in comparison to pristine graphene and reduced graphene [6]. Therefore, keeping the high variability of graphene in mind is important to understand the interaction, adsorption, transformation, and toxicity criteria of graphene.

## 3. Exposure, Accumulation, and Bio-Distribution of Graphene in Invertebrates

Invertebrates are important organisms utilized for environmental toxicological studies. They have a relatively short life span, fast reproduction, and high sensitivity towards pollutants. They are also cost-effective and contribute quick results. These organisms are regarded as convenient test species for ecotoxicity studies of new chemicals and nanoparticles [73]. The invertebrates enter the food chain at intermediate levels and are considered vehicles for recycling pollutants deposited in sediments. They feed on bacteria, plants, algae, and other invertebrates. These also become prey for larger organisms including fish and birds, which in turn represent a good portion of the human diet. Therefore, the fate of contaminants, bioconcentration, bioaccumulation, and biomagnification should be regarded as important platforms in understanding the toxicity produced, if there is any, over other species [74,75,76]. In a short-term study of 72 h on the eastern oyster (*Crassostrea virginica*), both 1 and 10 mg/L GO exhibited a dose-dependent elevated lipid peroxidation. No significant changes in glutathione S-transferase (GST) levels were observed, but a reduction in the total protein levels was found in tissues of the digestive glands at both concentrations (1 and 10 mg/L) of the GO. Overall, the study indicated that short-term GO exposures can induce oxidative stress, epithelial inflammation and adversely affected the overall health of *Crassostrea virginica* [77]. In a similar long-term study of 14 days on *Crassostrea virginica,* elevated lipid peroxidation and changes in glutathione-s-transferase (GST) were observed in tissues of gills and digestive gland exposed at 2.5 and 5 mg/L. Reactive oxygen species (ROS) induced oxidative damage. Therefore, the study suggested that detoxification enzyme such as GST are thought to be involved in stress signaling, leading to harmful effects on cellular health [78].

Further in a study by De Marchi et al., different concentrations of GO (0.01, 0.10, and 1.00 mg/L) toxicity were tested over *Diopatra neapolitana* (a polychaete species) for 28 days. The analysis of physiological (regenerative capacity) and biochemical (energy reserve, metabolic activity, and oxidative stress-related biomarkers) depicted negative effects of GO on regenerative capacity of *Diopatra neapolitana*, the organisms exposed to higher concentrations of GO took long periods to complete regeneration. GO also altered the energy-related responses, such as glycogen content with high polychaetes, which might have resulted from decreased metabolism. The research work concluded that cellular damage happened despite higher activities of antioxidant and biotransformation enzymes in *Diopatra neapolitana* exposed to GO [79]. In another study by Zhang and co-workers, acute and chronic toxicity tests were performed on *Daphnia magna* with and without the presence of humic acid (HA). The GO induced significant toxicity to *Daphnia magna* with a median lethal concentration of 48 h LC50 equal to 84.2 mg/L and 21-day LC50 as 3.3 mg/L. The HA mitigated the acute and chronic toxicity in *Daphnia magna*, as in the presence of HA, the decreased toxicity of GO was attributed to alleviation of oxidative damage by HA [80]. Furthermore, assessment of the accumulation and elimination of graphene after 24 h exposure to 250 μg/L of graphene in *Daphnia magna* was done by Guo and colleagues. Their results demonstrated the accumulation of graphene at 1% of organism’s dry mass. During the time for depuration, these organisms bore roughly constant body burdens after 24 h regardless of initial graphene concentration, whereas, after addition of an algae and humic acid, the depuration resulted in significant release of accumulated graphene but leaving some graphene in the organism. The study concluded that the remaining graphene in *Daphnia magna* might possess a risk to be passed on to neonates and thus require further risk assessment and relevant evaluation [81]. In another study by Souza et al., the toxicity of GO was further tested on freshwater flea *Ceriodaphnia dubia* in acute and chronic assay. The mean effective concentration (EC50) estimated during acute exposure was 1.25 mg/L, whereas chronic exposure of GO resulted in significant decrease in number of neonates, feeding rates, and ROS generation. Their research group concluded that in the presence of GO, there was a shift in the available energy for self-maintenance instead of feeding and reproduction activities [82]. Moreover, de Melo and co-investigators reported that when toxicity of GO was ascertained in presence and absence of trace elements (Cd, and Zn) in shrimp *Palaemon pandaliformis*, results suggested that GO did not cause acute toxicity at concentration up to 5.0 mg/L after 96 h exposure. However, GO in association with Cd and Zn increased toxicity of the trace elements as suggested by 96 h LC50 of Cd associated with GO 1.7 times less than the 96 h LC50 of Cd alone and 96 h LC50 of Zn associated with GO 1.8 times less than 96 h LC50 of Zn alone. Likewise, co-exposure of GO with trace elements impaired the routine metabolism of *Palaemon pandaliformis.* The study recommended that more researches and evaluation of data need to be done to understand the toxicity criteria of GO in aquatic organisms or aquatic bodies [83].

## 4. Exposure of Graphene to Fish Cell, In Vitro

In determining the cytotoxicity of environmental pollutants, cell lines derived from fish are considered very suitable experimental model. Testing the toxicity of nanomaterials is more complex than other chemical compounds. In nanomaterials, various physiochemical properties such as size, shape, surface area, and surface modification need to be considered prior to experimentation. Other factors including agglomeration, aggregation, sedimentation, dissolution, concentration need to be studied as well their fate in the test systems. If these aspects of nanomaterials are not investigated prior to experimentation, they might interfere with the results and misleading conclusions can be drawn. A study by Lamel et al. was done by exposing topminnow fish hepatoma cell line PLHC-1 cells to a single layer GO and carboxyl graphene (CXYG) (16 µg/mL GO or CXYG) suspensions with an aryl hydrocarbon receptor (AhR). Their results demonstrated pre- and co-exposure of cells to GO and CXYG nanoplatelets induced cytochrome P450 1A (cyp1A) expression, suggesting that graphene nanoplatelets increased the effective concentration of AhR agonists by facilitating passive diffusion into cells by damaging the plasma membrane or transporting them over the plasma membrane via a Trojan horse-like mechanism. Based on their results, it was found that there is an existence of combination effects between nanomaterials and environment pollutants [84]. In a similar study, when nanoplatelets GO and CXYG were assessed in PLHC-1 cells (GO: 0.125–16 g/mL; CXYG: 0.25–32 g/mL) for 72 h, oxidative stress was induced. Graphene nanoplatelets penetrated the plasma membrane and accumulated in cytosol, and these nanoplatelets interacted with the mitochondria and nuclear membrane further. The PLHC-1 also demonstrated reduction in the mitochondrial membrane potential and increase in ROS at 16 g/mL [85].

In another study, bluegill sunfish (BF-2 cells) were exposed to GO at concentrations of 0, 10, 20, 40, 60, 80, and 100 μg/mL for 24 h. Two biological assays (3-(4,5-dimethylthiazol-2-yl)-2,5-diphenyl tetrazolium bromide) MTT assay and neutral red uptake (NR) resulted in cytotoxicity and oxidative stress in BF-2 cells. The tested biomarkers of oxidative stress such as lipid peroxidation, superoxide dismutase, catalase, reactive oxygen species, and 8-hydroxy-2′-deoxyguanosine levels increased, and glutathione level was decreased in BF-2 after treatment with GO. The GO induced a dose- and time-dependent cytotoxicity on BF-2 cells [86]. In addition, using two different fish cell lines PLHC-1 and carp leukocytes (CLC), the toxicity and intracellular fate of graphene oxide (GO) were evaluated at a concentration range of 0–200 µg/mL for 24 and 72 h. The results depicted that GO had low cytotoxicity and is present in the vesicles as well as free in the cytoplasm of both cell types as revealed by transmission electron microscopy (TEM) [87].

## 5. Exposure, Accumulation, and Bio-Distribution of Graphene in Fish Embryo and Larvae

A reliable way of nanomaterials administration to an aquatic organism is oral and direct injection [88]. For direct injection, the vaccines/or any compound of interest are required to be prepared with oil/water formulations that may cause adverse effects. On the other hand, graphene nanomaterials are administered in fish using either by microinjection or by continuous exposure [89]. After careful selection, viable and fertilized eggs were either microinjected with graphene nanomaterials in nanoliter volumes within 4 hpf (hour post-fertilization) or continuously exposed in a medium containing graphene nanomaterials from 2 hpf until 72–168 hpf with change of medium once every 12–24 h interval [90,91,92,93]. The aggregation and stability of graphene nanomaterial can alter its physiochemical dimensions such as size and effective surface area, which may modulate toxicity to aquatic animals including fish [88]. The zebrafish embryos are covered by transparent acellular membrane called chorion, which acts as primary barrier that prevents the entry of exogenous materials including graphene nanomaterials from external environment in embryo’s body [65,94]. The route of graphene exposure, accumulation, and bio-distribution has been investigated previously. In a study to test the toxicity of GO and multi-walled carbon nanotubes (MWNTs), the results showed that GO has moderate toxicity to organisms as it inhibited growth and caused slight hatching delay among zebrafish embryos at 50 mg/L but did not depict significant increase on apoptosis in the embryo, whereas MWNTs’ toxicity demonstrated strong inhibition of cell proliferation and serious morphological defects in developing embryos even at lower dose of 25 mg/L. This particular work suggested that the distinctive toxicity of GO and MWNTs can be attributed to the different models of interaction between nanomaterials and organisms, which might arise from different geometric structure of nanomaterials [95]. In a similar study, when toxicity of GO, MWCNTs, and reduced graphene oxide (rGO) were tested on zebrafish embryos at a concentration of 1, 5, 10, 50, and 100 mg/L for 96 h, results revealed the inhibition of hatching of zebrafish embryos. The heart rate of the embryos treated with GO was significantly (*p* < 0.05) decreased at 100 mg/L at 48 hpf. Furthermore, rGO and MWCNTs decreased the length of hatched larvae at 96 hpf but no mortality and morphological malformation were observed, which further requires more results and analysis at various concentrations at different time periods [96].

Further in a different study, when the zebrafish embryos were exposed to a concentration of 1–100 μg/L to analyze the developmental toxicity, the results yielded impaired DNA modification, protein carbonylation, and excessive generation of ROS. The work also highlighted nonmonotonic response of zebrafish developmental toxicity to GO at μg/L to mg/L levels. On further analysis, transcriptomics revealed deficiencies in the collagen and matrix metalloproteinase (MMP) related genes, which in turn affected the skeletal and cardiac development of zebrafish. Moreover, to contribute to the results of developmental toxicity, metabolomics assay showed inhibition in amino acid metabolism and disturbance in the ratio of unsaturated fatty acids (UFAs) to saturated fatty acids (SFAs). The work, therefore, demonstrated developmental toxicity on the basis of involved molecular mechanism at trace concentration equal to 10 µg/L [60].

In an interesting study of Hu et al. with parent and offspring zebrafish when GO nanosheets were administered to parental zebrafish at a concentration of 0.01–1 µg/L, GO translocated from water to the brain of parent fish and the offspring with major loss of claudin5a (a core component of neuroepithelial barrier system). However, GO did not induce neurotoxicity in the parent fish and significant neurotoxicity occurred in the offspring, exhibiting loss of dopaminergic neurons and reduction in acetylcholinesterase (AChE) activity. Moreover, endoplasmic reticulum damage, autophagy promotion, ubiquitin down-regulation, and increase in β-galactosidase activities were observed, attributing to the failures in the carbohydrate and fatty acid metabolisms. Their work suggested that more researches and data compilation for the toxicity of GO on fish offspring at environmentally relevant concentration are required to do reliable analysis and to establish conclusions [97]. Similarly, in another study when the toxicity and molecular mechanism of GO were investigated in larvae and adult zebrafish at 0, 0.25, 0.5, and 1 mg/L for 72 h, results demonstrated that hepatotoxic phenotype has significantly decreased liver area and a dose-dependent decrease in the number of hepatocytes. Moreover, the number of macrophages and neutrophils in zebrafish embryos was reduced, but the expressions of pro-inflammatory cytokines were increased after GO treatment. The thorough analyses revealed down-regulation of lipid metabolism genes and up-regulation of immune genes. Moreover, GO induced NF-κB p65 into the nucleus and increased the protein levels of NF-κB p65, JAK2, STAT3, and Bcl2 in adult zebrafish liver. Overall, the elaborative study and results demonstrated GO-induced hepatic dysfunction mainly through the ROS and PPAR-α-mediated innate immune signaling in zebrafish [98].

In a study by Clemente and co-workers, zebrafish embryos were analyzed after 5 and 7 days of GO exposure at 100 mg/L and humic acid (HA) at 20 mg/L either individually or together. Regardless of the presence of HA, the larvae exposed to GO for 5 days demonstrated increase in the locomotor activity, reduction in yolk sac size, total length, and inhibition of AChE activity. The larvae exposed to GO for 7 days did not show any significant differences in the locomotor activity, but the RT-PCR gene expression analysis depicted an increase in AChE expression. The work suggested that HA is associated with toxicity risk modulation by GO and some compensatory homeostatic mechanisms might be involved in the effects observed in zebrafish [99]. In a similar study when GO and HA were exposed to zebrafish embryos at 0–100 mg/L, significant hatching delay and cardiac edema were observed. The interaction of GO with chorion induced damage to chorion protuberances, excessive generation of ^∙^OH, and changes in the secondary structure of protein, which, in contrast, were relieved by HA. On the other hand, humic acid reduced interactions between GO and chorion mitigating chorion damage by regulating the morphology, structure, and surface negative charges of GO, indicating a feasible antidotal mechanism for GO in presence of HA [100].

In another study, zebrafish embryos were treated with GO at 0, 0.01, 0.1, 1, and 100 mg/L for 24, 48, and 96 hpf. Results revealed that the GO adhered to and enveloped the chorion of zebrafish embryos via the hydroxyl group interactions and it blocked the pore canals of the chorionic membrane causing hypoxia and hatching delay. Furthermore, GO penetrated the chorion of embryo via endocytosis, damaged the mitochondria, and primarily translocated to the eye, heart, and yolk sac regions involved in circulatory system of zebrafish. In these organs, GO induced excessive generation of reactive oxygen species, thus increasing oxidative stress, DNA damage, and apoptosis. The work highlighted specific adverse effects of GO on embryogenesis and emphasized the potential ecological and health risks of GO [101]. Furthermore, when GO exposure was done to zebrafish embryos at 5, 10, 50, or 100 mg/L for 6 days and up-regulation in *synapsin IIa* expression at 5 mg/L with down-regulation of *dat* expression were observed, showing potential compensatory mechanism. At 10 mg/L GO exposure, increase in heart rate, absolute turn angle, brain cell damage, and decrease in dopamine levels were observed. However, no changes were observed on higher concentrations of GO exposure, which is attributed to GO agglomeration. The work concluded that the results of the toxic effects of GO were not dose-dependent and are pre-eminent at lower concentrations, and hence more studies are required to gather relevant data and analysis [102]. In the next study, dose-dependency effects of three different sized GO particles (50–200 nm, <500 nm, and >500 nm) on zebrafish during the very early developmental stages (4–124 h post-fertilization) were performed. The GO nanoparticles accumulated in the eyes, the heart, the yolk sac, and the blood vessels of the fish larvae. This was also exhibited on observed endpoints of delay in hatching time, shortened body length, modification in heart rate and blood flow, response in photoperiod stimulation, enhanced activities of superoxide dismutase, AChE, caspase-3, and induction of apoptosis-related genes expression. Specifically, oxidative stress and induction of apoptosis in all three different sized GO particles predicted a potential risk of GO on marine organisms [103]. The group of Li reported that when corannulene (non-planar PAH) and graphene (planar PAH) were exposed to zebrafish larvae at 1, 10, and 50 µg/mL, minimal developmental toxicity and significant decrease in locomotion/increase in sleep caused by corannulene with no significant locomotion alterations at 50 µg/mL graphene were observed. Corannulene increased sleep and reduced locomotor activity and the expression of *hcrt* and *hcrtr* mRNA, while graphene did not obviously disturb the sleep behavior and gene expression patterns [60].

## 6. Exposure, Accumulation, and Bio-Distribution of Graphene in Adult Fish

Acute toxicity, oxidative stress, and immune-toxicity of GO were analyzed at 1, 5, 10, or 50 mg/L for 14 days were done in a study using the zebrafish adult model. Histological analysis of the liver and intestine showed cellular alterations including vacuolation, loose arrangement of cells, histolysis, and disintegration of cell boundaries. Malondialdehyde (MDA) level and superoxide dismutase (SOD) and catalase (CAT) activities were increased and glutathione was decreased in the liver after treatment with GO. Moreover, levels of tumor necrosis factor alpha (TNF-α), interleukin-1B, and interleukin-6 depicted induction in immune response. Hence, the work suggested GO exposure in aquatic system caused oxidative stress and immune toxicity in adult zebrafish [104]. Moreover, the adult zebrafish was exposed to carbon-14-labeled few-layer graphene (FLG) to analyze the effect of size on graphene uptake, depuration, and bio-distribution as reported by Lu et al. in their research article. After 48 h exposure to larger FLG (L-FLG) at 250 μg/L, the amount of graphene in the organism was close to 48 mg/kg fish dry mass, which was more than 170-fold greater than the body burden of those exposed to the same concentration of smaller FLG (S-FLG). The L-FLG mainly accumulated in the gut of adult zebrafish, and S-FLG was observed in both the gut and the liver after exposure with or without NOM. Strikingly, the S-FLG was able to pass through the intestinal wall and enter the intestinal epithelial cells and blood. The work suggested further tests to contribute concrete results [105]. In another study, zebrafish were fed diets with three graphene family materials (GFMs) monolayer graphene powder (GR), graphene oxide nanosheet (GO), and reduced graphene oxide powder (rGO) for 21 days. Later, gut bacterial communities were analyzed and depicted alteration in intestinal morphology and oxidative stress. Results showed that GFMs led to different inflammatory responses and significantly altered the relative composition of the gut bacterial species by increasing the relative abundance of *Fusobacteria* and the genus *Cetobacterium* and *Lactobacillus* and decreasing the abundance of Firmicutes and the genus *Pseudomonas*; GR caused marked shifts in the diversity of the gut microbiota. The GFMs also altered the intestinal morphology and antioxidant enzyme activities by inducing more vacuolation and generating more goblet cells [106].

**Figure 2:**
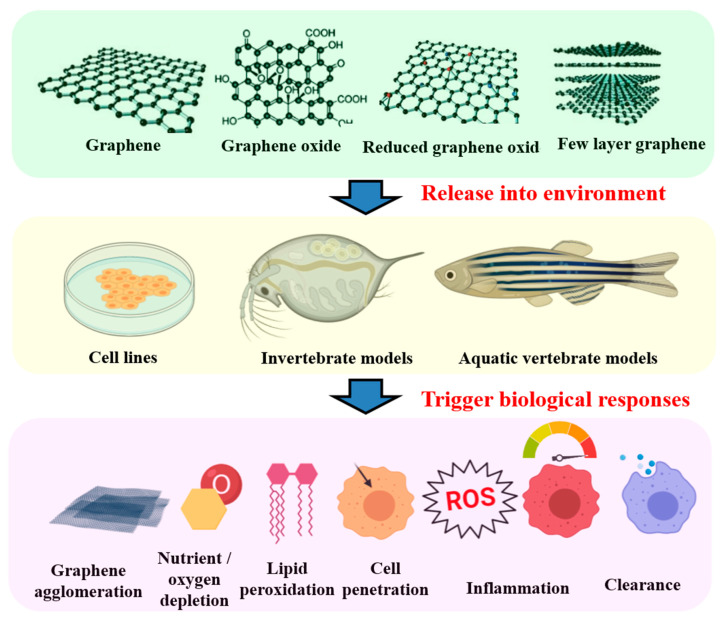
Current understanding of the potential toxicity induced by graphene-based nanomaterials graphene, graphene oxide, reduced graphene, and few-layer graphene are listed in the top panel (green color) in fish cells, invertebrates, embryo, and adult aquatic organisms. The common-used in vitro and in vivo aquatic animal models are listed in the middle panel (yellow color). The potential cytotoxicity induced by graphene-based nanomaterials is summarized in the bottom panel (pink color).

The effects of GO were observed on the antioxidant metabolism of zebrafish; after 48 h exposure, SOD activity was significantly increased in 20 mg/L, CAT activity in 2, 10, and 20 mg/L, and lipid peroxidation (LPO) had an increase in 2 mg/L, whereas glutathione peroxidase (GPx) was inhibited at 20 mg/L. Later after 168 h recovery in clean water, SOD remained elevated in 20 mg/L, CAT activity remained unchanged and GPx activity was inhibited at 2, 10, and 20 mg/L, and LPO decreased in 2 mg/L. The study suggested that GO exposure disrupted antioxidant metabolism of adult zebrafish in which it was not restored even after 168 h recovery period in clean water [107]. The toxic effects of GO when evaluated on *Anabas testudineus* (the climbing perch) revealed accumulation of cellular lipid peroxides specifically in the mitochondria. Activity of SOD, CAT, and, GST was augmented in contrast to lowered level of reduced glutathione titer. The results also indicated oxidative stress in cell and mitochondria in fish after exposure to GO, thus suggesting compilation of more data for comparison of results and establishments [108]. In a separate study with graphene nanoparticles, fish were exposed in 10, 20 mg/L concentrations for 10 days. Results of statistical analysis showed significant decrement at *p* ≤ 0.05 for RBC (0.770 cells/μL × 10^6^) in T1 and 0.850 cells/μL × 10^6^ in T2 compared with negative control (1.410 cells/μL × 10^6^). There was no significant decrease *p* > 0.05 of each PCV in T1 and T2 (31 and 27%), respectively, compared with control negative (35%) and hemoglobin (10.30 and 9 mg/dL) in T1 and T2, respectively, compared with negative control (11.65 mg/dL), while the addition of graphene nanoparticles did not affect the number of WBC in T1 and T2 (15.1 and 19.1 cells/μL × 10^3^), respectively, compared with negative control (15.6 cells/μL × 10^3^) [109]. The summary of graphene and GO-related toxicity in aquatic animals have been compiled in Table 1 and depicted in Figure 2 in the following section.

## 7. Current Understanding of Graphene and Graphene Oxide (GO) Toxicity and Knowledge Gaps along with Other Carbon Nanomaterials

The unique properties of graphene and GO along with other carbon nanomaterials such as carbon nanotubes and fullerenes have been used for various applications and can be released in environment at significant amounts, posing threat to aquatic species as well as humans. Once released in the bodies of water, graphene and GO can also interact with existing inorganic ions and natural organic matter (NOM), posing threat for significant adverse effects on the ecosystem. However, intentional exposure of graphene in biomedical applications is of interest. The literature cites approximately 60% graphene-related materials applications in biomedical engineering as stated by Mao et al. [110]. This signifies scientific community is focused on developing various forms of therapy with graphene-based nanomaterials, which may yield commercialized products in the market soon [111]. Moreover, GO could be modified to have cell surface receptors that act like a net to reduce endocytosis and starve cells to death. This modification of GO with an anti-cancer drug might provide additive effects to cancer cell killing in BEA-2B and KB cervix tumor cells after 48 h via WST-8 [112]. Hence, it becomes important to analyze the toxicity criteria of graphene-related nanomaterials to help understand the specific mechanism of their working with various modifications, dose, physiochemical properties, and model organisms. The information regarding overall concentration of graphene and GO being released in aquatic environment is unknown [65]. However, the concentration of engineered nanomaterials based on probabilistic material free computer model ranges from ng/L to µg/L [113]. Consequently, the disposition of graphene and GO in aqueous environment is studied under this range. The published studies are still inadequate to establish guidelines for environmentally relevant concentration of graphene and GO to set limits for safety measurements of aquatic organisms.

**Figure 3:**
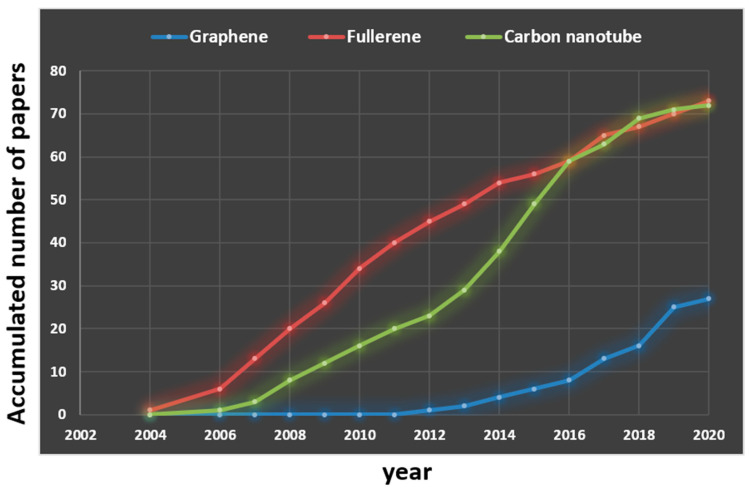
Comparison of paper publication number related to aquatic toxicity induced by three carbon-based nanomaterials during the past 16 years (from 2004 to 2020). Papers relevant to graphene-based nanomaterials are highlighted with blue color. Papers relevant to fullerene-based nanomaterials are highlighted with red color. Papers relevant to carbon nanotube are highlighted with green color.

Here in the present review, we have compiled, presented, and compared the current toxicological scenarios of graphene and GO in aquatic invertebrates and fish model organisms. We compared the paper published number (regarding aquatic toxicity induced by carbon-based nanomaterials) during the past 16 years (from 2004 to 2020). Results show the relative paper published number for graphene-based nanomaterial toxicity is around 50% less than their fullerene or carbon nanotube counterparts (Figure 3). In addition, during the compilation of this review paper, it has been observed that there exist plenty of knowledge gaps of carbon nanomaterials (graphene, carbon nanotubes, and fullerenes) specifically on organ toxicity of model organisms. Most of previous studies only address LC50 or whole-body enzyme activity alterations after carbon-based nanomaterial exposure. Although carbon-based nanomaterials toxicity studies in mentioned aquatic organisms have been evaluated in some limited dimensions, there exists a huge gap in understanding of organ toxicity of these organisms. However, the most prominent findings studies have reported are oxidative stress, lipid peroxidation, and cellular penetration; there is lack of existing studies on whole organism level like behavioral, transcriptomic, and histopathological analysis.

In case of continuous waterborne exposure, the developmental stages of the embryo and the duration of exposure are very critical. The embryos exposed in waterborne graphene and GO might agglomerate in the exposure media or on the chorion depleting embryo of oxygen supply and may cause developmental deformities or worst, mortality. The chorion has been reported as a major barrier to prevent nanomaterial uptake by fish embryos [114]. In this consideration, microinjection can be used to deliver nanomaterials to the developmental embryos [60,98]. However, in this particular treatment, the injected nanomaterials will be eventually diluted over time with the rapidly dividing embryonic cells. For adult fish, the most popular method for graphene delivery is waterborne exposure. However, the insoluble nature of graphene makes it difficult to measure the exact delivery dose. Other more precise delivery methods like oral delivery can be considered in the future [115]. Hence, to establish the toxicity criteria further, more studies are considered necessary to understand how graphene and GO can induce toxicity. As for the data available for analysis, it is difficult to set safety limits and differentiate in the effects caused by tested nanomaterials whether actual or in waterborne concentration on the organisms.

## 8. Summary

In this review paper, we hereby summarize that research of graphene and GO toxicity specifically on aquatic organisms is relatively limited. The research on graphene-based nanomaterials toxicity has started taking place progressively in the last decade. Thus, a comprehensive understanding of interaction of graphene and GO with environment, aquatic organisms, and other living systems in vitro and in vivo is essential for their safe usage and further development. The large part of available current literature indicates that graphene-based nanomaterials are cytotoxic. Although the particular mechanism for their toxicity has not yet been established, ROS elevation, lipid peroxidation, nutrient/oxygen depletion, and inflammation have been most widely recognized mechanisms for graphene-based nanomaterials toxicity in aquatic organisms.

The applications and uses of graphene and GO are rising evidently in fields of biomedical sciences, supercapacitors, sensors, and construction materials. The release of waste material from these industries can prove to be harmful for environment, aquatic organisms, and humans. Graphene-based nanomaterials interact with natural organic material (NOM), aggregates, adsorbs, and colloids, which in turn can cause toxicity to aquatic organisms through many different body mechanisms. Furthermore, the physiochemical properties of graphene and GO such as particle size, surface functional groups, and oxygen content/surface charges may affect the toxicity upon interaction with aquatic organisms. The data we accumulated demonstrated a lot of information gaps that does not allow establishing a concrete statement regarding the toxicity criteria guidelines. It is also difficult to compare the toxicological effects of graphene and GO between different studies due to diversity in size, shape, surface modification, synthetization techniques, and model organisms. It is, hence, important to understand the toxicity caused by graphene nanomaterials for aquatic organisms in order to facilitate the practical applications of these promising new graphene-based nanomaterials.

## Figures and Tables

**Table 1:** Summary of graphene and graphene oxide (GO) toxicity in aquatic animals.

Animal	Route of Graphene Exposure	Adverse Outcome	Dosage Concentration and Time	Ref.
Tested in invertebrate species				
*Crassostrea virginica*	Waterborne exposure	Short-term GO exposures can induce oxidative stress, epithelial inflammation, and adversely affect overall *Crassostrea virginica* health.	1 and 10 mg/L 72 h static renewal.	[77]
*Crassostrea virginica*	Waterborne exposure	Elevated lipid peroxidation and changes in glutathione-s-transferase (GST) activities were observed in gills and digestive gland tissues of the GO-exposed oysters. Oxidative damage, stress signaling leading to adverse effects on cellular health.	2.5 and 5 mg/L 14 days	[78]
*Diopatra neapolitana*	Waterborne exposure	GO induced negative effects on the regenerative capacity, altered energy-related responses, especially glycogen content, and decrease in metabolism, cellular damage in *Diopatra neapolitana*.	0.01, 0.10 and 1.00 mg/L 28 days	[79]
*Daphnia magna*	Waterborne exposure	GO induced significant toxicity to *Daphnia magna*. 21 days LC50 chronic toxicity 3.3 mg L^−1^. In the presence of HA, the decreased toxicity of GO was attributed to the alleviation of oxidative damage by HA.	50.0, 65.0, 84.5, 110.0 and 143.0 mg/L 21 days	[80]
*Daphnia magna*	Waterborne exposure	^14^C-labeled graphene accumulated 1% of the organism dry mass. Excretion of graphene at constant phase in depuration. Addition of algae and humic acid to water during the depuration period resulted in release of a significant fraction (~90%) of the accumulated graphene, some remained in the organism. Accumulated graphene in adult *Daphnia* was likely transferred to the neonates.	250, 100, 50 and 25 µg/L 48 h Depuration 24 h	[81]
*Ceriodaphnia dubia*	Waterborne exposure	GO induced lethality, reproduction inhibition, ROS generation, reduction on feeding rates and accumulation on gut tract. There was a shift in the available energy for self-maintenance rather than feeding or reproduction activities.	Acute exposure: 0.1; 0.2; 0.4; 0.8; 1.6 and 3.2 mg/ L, 48 h Chronic exposure: 0.05; 0.1; 0.2; 0.4 and 0.8 mg/ L 7 days	[82]
*Palaemon pandaliformis*	Waterborne exposure	GO did not present acute ecotoxicity at concentrations up to 5.0 mg/L. The 96 h LC50 of Cd associated with GO was 1.7 times less than the 96 h LC50 of Cd alone and the 96 h LC50 of Zn associated with GO was 1.8 times less than the 96 h LC50 of Zn alone. The co-exposure of GO with trace elements impaired the routine metabolism of *Palaemon pandaliformis*.	GO - 0.1; 1.0; 2.5 and 5.0 mg/L 96 h Co-exposure of GO 1.0 mg/L with trace elements Cd 1.0 mg/L and Zn 1.0 mg/L	[83]
*Cyprinus carpio L.*	Waterborne exposure	Significant decrease in RBC count. No significant effect on WBC, PCV, and Hb.	0, 10, 20 mg/L, 10 days	[109]
Tested in fish cell lines				
PLHC-1	Co-exposure - increasing concentration of AhR agonist alone or in presence of GO and CXYG. Pre and post exposure – increasing concentration of BKF and α-MEM, to α-MEM + 4 mg/mL CXYG or to α-MEM + 16 mg/mL CXYG for another 24 h	GO and CXYG had potentiating effect on PAH- and PCB-induced Cyp1A expression at both the transcriptional and the enzymatic levels. It suggested surface chemistry of GO and CXYG did not had influence on the direct or indirect interaction with the selected AhR agonists. The obtained results suggest that a preceding and/or simultaneous exposure to GO or CXYG nanoplatelets may modify the toxicokinetics of aromatic environmental pollutants such as PAHs and PCBs.	GO and carboxyl graphene (CXYG) at 16 µg/mL, AhR agonist.	[84]
PLHC-1	α-MEM medium	PLHC-1 cells demonstrated significantly reduced mitochondrial membrane potential (MMP) and increased ROS levels at 16 μg/mL GO and CXYG (72 h), but barely any decrease in cell viability. The observation of intracellular graphene accumulations not enclosed by membranes suggests that GO and CXYG internalization in fish hepatoma cells occurs through an endocytosis-independent mechanism.	GO: 0.125–16 µg/mL; CXYG: 0.25–32 µg/mL	[85]
BF2	GO in milli Q water (stock solution) + Eagle’s medium	GO caused mitochondrial and lysosomal damage to BF-2 cells, oxidative stress, and morphological changes by GO through ROS, as indicated by the evaluated biomarkers LPO, GSH, SOD, CAT, and 8-OHdG.	0, 10, 20, 40, 60, 80 and 100 μg/mL for 24 h	[86]
PLHC-1 and CLC	GRMs – Carbon nanofibers (CNFs) and graphene oxide (GO)	GO sheets were present within vesicles as well as free in the cytoplasm of both cell types. CNFs toxicity was inversely related to the graphitization degree.	0–200 μg /mL of GRMs for 24 and 72 h	[87]
Tested at embryonic or larvae stages of fish				
*Danio rerio*	Waterborne exposure	Hatching delay of zebrafish embryos at a high dosage of 50 mg/L. Embryos exposed to GO exhibited significant cellular apoptosis only in the forehead and eye region, and no aggravation of cellular apoptosis was observed with increasing concentration of GO.	0, 3.4, 7.6, 12.5, 25 and 50 mg/L 96 h post fertilization	[95]
*Danio rerio*	Waterborne exposure	GO impaired DNA modification, protein carbonylation, ROS generation (also superoxide radical)	1–100 µg/L 2.5 hpf-7 dpf	[60]
*Danio rerio* (adult and embryo)	Waterborne exposure	GO translocated from the water to the brains of parental and offspring fish with a significant loss of *claudin5a.* GO did not trigger obvious neurotoxicity in parental zebrafish, whereas remarkable neurotoxicity occurred in the offspring, which exhibited a loss of dopaminergic neurons and reductions in acetylcholinesterase activity.	GO exposed to parental zebrafish 24 h prior mating 0.01–1 μg/L	[97]
*Danio rerio*	Waterborne exposure	Regardless of the presence of HA, larvae exposed to GO for 5 days showed an increase in locomotor activity, reduction in the yolk sac size, and total length and inhibition of AChE activity, but there was no difference in enzyme expression. Results indicated that HA is associated with the toxicity risk modulation by GO.	GO-100 mg/L & HA 20 mg/L alone or together for 5–7 days	[99]
*Danio rerio*	Waterborne exposure	GO adhered to and enveloped the chorion of zebrafish embryos mainly via hydroxyl group interactions, blocked the pore canals of the chorionic membrane, and caused marked hypoxia and hatching delay. GO induced excessive generation of reactive oxygen species and increased oxidative stress, DNA damage, and apoptosis	0, 0.01, 0.1, 1, 100 mg/L for 24, 48 and 96 hpf	[101]
*Danio rerio*	Waterborne exposure of GO, Humic Acid (HA) and GO-HA	GO induced significant cardiac edema and hatching delay. HA decreased the interaction between GO and chorion, mitigated chorion damage by regulating morphology, structures, and surface negative charges of GO	GO 0–100 mg/L HA 0–100 mg/L 2.5 hpf-72 hpf	[100]
*Danio rerio* (larvae and adult)	Injections at ventral end of larvae	GO induced hepatic dysfunction through the ROS and PPAR-α mediated innate immune signaling in zebrafish	0, 0.25, 0.5, and 1 mg/L for 72 h	[98]
*Danio rerio*	Waterborne exposure of GO and reduced graphene (rGO)	GO had significant effects on the heart rate, while rGO affected the embryos hatching and the length of larvae in a dose-dependent manner	1, 5, 10, 50, 100 mg/L for 96 h	[96]
*Danio rerio*	Waterborne exposure	GO induced cardiac and dopaminergic alterations, as well as neuronal gene expression and morphology modifications. Altered locomotion in terms of increase of turn angle suggesting parkinsonian-like motor symptoms (at low concentrations).	5, 10, 50 or 100 mg/L for 6 days	[102]
*Danio rerio*	Waterborne exposure	GOs induced oxidative stress and apoptosis. In particular, the immune cell number, pro-inflammatory iNOS activity, and AChE activity (a neural development indicator) were found to be induced to some extent after GO exposure, suggesting the presence of both immunomodulatory and neurotoxic effects in zebrafish larvae. The waterborne-GO exposure on zebrafish during early development was not merely dependent on GO concentration but also the associated GO sizes.	GO particles (50–200 nm, <500 nm, and >500 nm) at 0.1, 1, 10, and 100 mg/L for 4–124 h post-fertilization	[103]
*Danio rerio*	Microinjection (4 nL/embryo)	Graphene induced no significant locomotion alterations, sleep behavior, and gene expression patterns.	Graphene at 1, 10, 50 µg/mL	[60]
Tested at adult stage of fish				
*Danio rerio*	Waterborne exposure	GO caused toxicity-Oxidative stress and tissue damage induced in fish by GO through ROS, indicated by the biomarkers of MDA, GSH, SOD, and CAT; GO caused immunotoxicity in fish indicated by increased expression of inflammatory cytokines, TNF-, IL-1, and IL-6.	0, 1, 5, 10 or 50 mg/L GO for 14 days	[104]
*Danio rerio*	Carbon 14 labeled few-layered graphene (FLG)	At 48 h larger FLG (L-FLG) at 250 µg/L the amount of graphene was close to 48 mg/kg fish dry mass, 170-fold greater than body burden of the same concentration of smaller FLG (S-FLG). L-FLG accumulated in gut and S-FLG accumulated in gut and liver. L-FLG and S-FLG had significantly different impact on intestinal microbial community structure.	L-FLG- 300–700 nm S-FLG-30–70 nm 50 μg/L, 75 μg/L and 250 μg/L 4, 12, 24, 48, 72 h	[105]
*Danio rerio*	Graphene family materials (GFMs), Monolayer graphene powder (GR), graphene oxide nanosheet (GO), reduced graphene oxide powder (rGO)	GFMs led to different inflammatory responses and significantly altered the relative composition of the gut bacterial species. GFMs altered the intestinal morphology and antioxidant enzyme activities.	1 µg in fish diet for 21 days	[106]
*Danio rerio*	Waterborne exposure	GO caused increase in oxidative stress, increase in lipid peroxidation, changes in SOD, CAT, GPx. After 168 h: GO toxic effects decreased, but homeostasis not fully recovered.	2, 10, and 20 mg/L 48 h, recovery period 168 h	[107]
*Anabas testudineus*	Injection at base of caudal fin	GO induced oxidative stress in cell and mitochondria in fish	200 µL from 1 g/L 100 mg/L, 10 mg/L of GO in aqueous solution 24 h	[108]
